# Reliability Assessment of Thermocompressed Epoxy Molding Compound through Glass via Interposer Architecture by the Submodeling Simulation Approach

**DOI:** 10.3390/ma15207357

**Published:** 2022-10-20

**Authors:** Shih-Hung Wang, Wensyang Hsu, Yan-Yu Liou, Pei-Chen Huang, Chang-Chun Lee

**Affiliations:** 1 Department of Mechanical Engineering, National Yang Ming Chiao Tung University, No. 1001, Ta Hsueh Rd. East Dist., Hsinchu City 30010, Taiwan; 2 Department of Power Mechanical Engineering, National Tsing Hua University, No. 101, Section 2, Kuang-Fu Road, Hsinchu 30013, Taiwan

**Keywords:** TGV, thermocompression, finite element analysis, submodeling technique

## Abstract

In glass interposer architecture and its assembly process, the mechanical responses of interposer structure under thermocompression process-induced thermal loading and generated shrinkage of molding material are regarded as a major reliability issue. Thousands of metal-filled via are involved in glass interposers and are regarded as a potential risk that can lead to cracking and the failure of an entire vehicle. In this study, a finite element-based submodeling approach is demonstrated to overcome the complexity of modeling and the relevant convergence issue of interposer architecture. Convergence analysis results revealed that at least four via pitch-wide regions of a local simulation model were needed to obtain the stable results enabled by the submodeling simulation approach. The stress-generation mechanism during thermocompression, the coefficient of thermal expansion mismatch, and the curing process-induced shrinkage were separately investigated. The critical stress location was explored as the outer corner of the chip, and the maximum first principal stress during the thermocompression process generated on the chip and glass interposer were 34 and 120 MPa, respectively.

## 1. Introduction

Among three-dimensional (3D) integration techniques of advanced electronic packaging, interposer architecture is regarded as a promising solution for achieving 3D integration with ultrafine pitch features. Interposer techniques based on mainstream semiconductor materials, such as silicon (Si), have been widely developed in accordance with the advantage of their compatibility with current semiconductor manufacturing processes. However, Si interposers have limitations in cost and performance, depending on the wafer size and usage ratio; moreover, they are limited by the poor electrical characteristics of Si itself. Accordingly, glass-based interposer technology has been demonstrated to serve as a replacement for Si interposers. Glass has relative superior electrical properties, a manageable coefficient of thermal expansion (CTE), low fabrication process cost, and prospective compatibility with next-generation panel fabrication processes [1,2,3]. Accordingly, glass-based interposers are regarded as the most promising material to replace the Si-based interposer technique; substantial literature has demonstrated the related technology, including the fabrication, thermal treatment, and metallization processes [4,5,6,7,8]. The embedded integrated circuit architecture with glass interposer has been demonstrated to meet the requirement of fine line/space and high-density interconnections [9]. Thin glass interposers with a fine via diameter and I/O pitch have been developed for specific applications for ultrahigh bandwidths [10]. From the viewpoint of mechanical reliability concern, the major stress-generation mechanism results from the CTE mismatch between the components in interposer architecture. The time- and temperature-dependent protrusion behavior of copper (Cu)-filled glass via (TGV) under different annealing temperatures and dwell times has been analyzed [11]. A specific failure of radial and circumferential cracks has been separately observed and attributed to the stress buildup during the heating and cooling process in the annealing heat treatment procedure [12,13]. The critical stress location of Cu-filled TGV has been explored as a Cu/glass interface because the mismatching of the aforementioned material CTE, the slower ramp rate, and the decent dwell time can help manage the thermal stress [14,15]. To reduce Cu protrusion-induced stress, a conformal-type TGV has been demonstrated, and a fully-filled TGV with considerable copper protrusion during thermal cycling was revealed [16]. The interfacial energy release rate of the interface between Cu-filled TGV and different glass materials has been investigated through the finite element analysis (FEA) simulation and analytical model [17]. For different filling materials of TGV, the crack formation mechanism can differ considerably; moreover, radial-type and circumferential-type cracks were found in electroplated Cu-filled TGV and sliver-paste-filled TGV during the heating process in [18]. The structural layout design factor of stress-to-size ratio was analyzed, and the analysis results showed that the Cu via thickness considerably affected the stress behavior in the glass interposer and should be carefully managed to reduce the stress and decrease the size of the package in [19]. Similar structures on both sides of the glass panel were preferred in order to minimize a process-induced warpage issue prior to assembly in [20]. The microfluidic characteristics of TGV during the reflow process were investigated by experimental work and demonstrated analytical model [21]. To study the critical mechanical characteristics of TGV, a novel sample preparation procedure of free-standing TGV pillar was demonstrated, and its mechanical properties were measured by the microcompression technique in [22]. The interactive influence of material and structural design of TGV architecture on thermal stress and crack driving force were effectively estimated by the FEA simulation in [23]. Thermal cycling and high-temperature storage test were performed on the glass interposer integrated with polymer-based dielectric material as passivation, and the tested results of the electrical resistance of the vehicle remained at the original value after thermal cycling in [24]. The fatigue life of Cu-filled via was demonstrated by the Coffin–Manson equation integrated with Engelmaier’s model, and contributed to the quantitative estimation of the mechanical reliability of the Cu via structure in [25]. Sometimes, the difference between experimental and simulation results are caused by the microstructure variation or defects contained in vehicle. Genetic algorithms and multi-response optimization are utilized in studies to optimize and analyze the residual stresses with time-independent cyclic plasticity in welding joints [26,27]. Multi-response optimization has also been used in hyper-elastic materials characterization [28]. Due to the Cu protrusion phenomenon introduced in the TGV interposer, an additional machine treatment might need to be performed. The relative modeling setting concept of the roller was demonstrated and improved in [29]. The contact behavior of the roller was comprehensively demonstrated by finite element method, experimental analysis and analytical models in [30,31]. Electrical–thermal responses of vias containing glass interposers, the temperature dissipation ability of Cu-filled via and carbon nano tube(CNT)-filled via were analyzed, and the CNT-filled via showed 30 °C lower temperature compared with the Cu-filled via in [32]. Different mechanical modeling approaches were utilized to investigate the solder joint reliability of TGV-interposer-based fan-out packaging in [33]. Warpage characterization of the glass interposer with chemically shrunk epoxy molding compound (EMC) were estimated through experimental and simulation work in [34]. Among the simulation work discussed was the mechanical response of TGV interposer architecture. Most studies researched the warpage behavior of an entire interposer, or estimated the stress induced by the annealing procedure on a TGV interposer by analytical solution. However, those studies did not reveal the stress-generation mechanism, and also overcame the complexity of the FEA mesh gridding issue of the TGV interposer with a significant size mismatch between components. Accordingly, this study demonstrated a process-oriented stress simulation approach integrated with the submodeling technique to analyze the mechanical reliability of the TGV interposer in the thermocompression fabrication procedure. The major purpose of the simulation methodology in the present study is to reveal how the roles in FEA modeling can obtain a stable and reliable simulation result without excessive consumption of computing resources. 

## 2. Structural Layout Design and Fabrication Process of Glass Interposer Architecture

**Figure 1:**
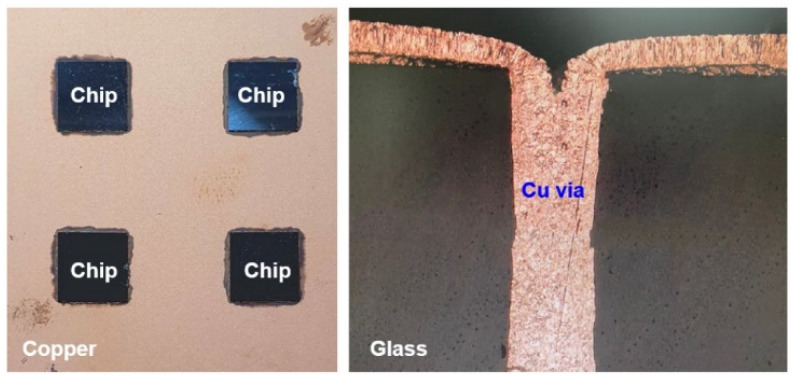
Illustration of mounted chip on TGV interposer and cross-sectional view of Cu-filled glass via.

Among current mainstream electronic packaging architectures, the epoxy molding compound is an important component, the chemical shrinkage characteristic of EMC is an additional stress-generation resource, and its influence on glass interposer architecture is not explored systemically. For this reason, this study is mainly focused on the mechanical responses of the glass interposer vehicle capped with thermocompressed EMC material. A diagram of the utilized TGV interposer is illustrated in Figure 1. The stress-generation mechanism introduced from the thermocompression procedure is investigated by the FEA submodeling technique, and the detailed analysis flow is described in the next section.

**Figure 2:**
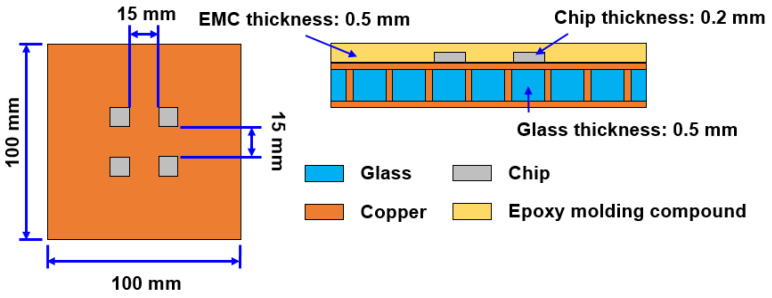
Schematic and structural parameters of glass interposer vehicle.

**Figure 3:**
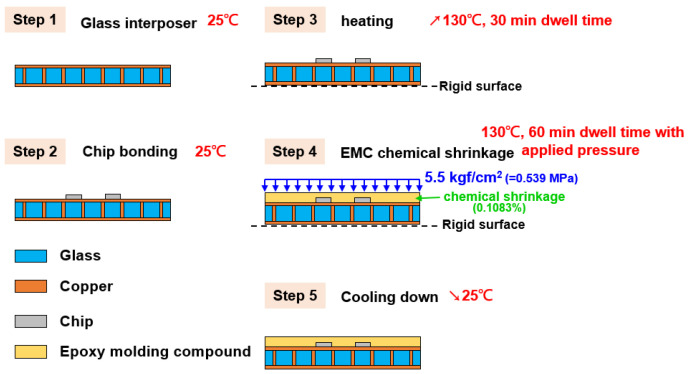
Fabrication process diagram of glass interposer with array-type and process conditions and material components.

As shown in Figure 2, the glass interposer vehicle has the following characteristics: 100 mm × 100 mm area, four mounted Si chips with 10 mm × 10 mm area, and a 15 mm gap between each chip; the thicknesses of the Si chip, glass interposer, and EMC are 0.2, 0.5, and 0.5 mm, respectively. The TGV of the glass interposer was designed with a 28-μm via diameter and a 1-mm via pitch. The fabrication process of the aforementioned vehicle is illustrated in Figure 3. The glass interposer with filled TGV was first prepared, and then the chip was mounted upward at room temperature (25 °C). Afterward, the glass interposer vehicle was placed upon the vacuum platform to prevent slides during the process. After checking the stability of the vehicle, the chamber was preheated up to 130 °C, and then a compression load of 5.5 kgf/cm^2^ (0.539 MPa) was applied to accomplish the curing process of the selected EMC. T_g_ of the selected EMC was 172 °C, so that the CTE of EMC could remain at the same approximate level as other components during the thermocompression process, especially the brittle Si chip and glass interposer. Finally, the applied compression load was removed, and the glass interposer vehicle was cooled down to room temperature to finish the entire fabrication process. The specific behavior of the selected EMC material, i.e., chemical shrinkage, was 0.1083% under fully cured conditions. This means the mechanical responses generated from the thermocompression process can be divided into two major parts: the CTE-mismatch induced thermal stress, and the stress introduced by the shrinkage of the EMC itself. Foregoing mechanical loadings are unfavorable for the reliability of glass interposers in accordance with the Si chip, and glass might have the risk of cracking failure occurrence. Accordingly, the finite element analysis methodology was utilized in this study to reveal the influence of the thermocompression process on glass interposer vehicles.

## 3. FEA Modeling of Global Glass Interposer Architecture and Local TGV Array Region Model Based on the Submodeling Technique

**Figure 4:**
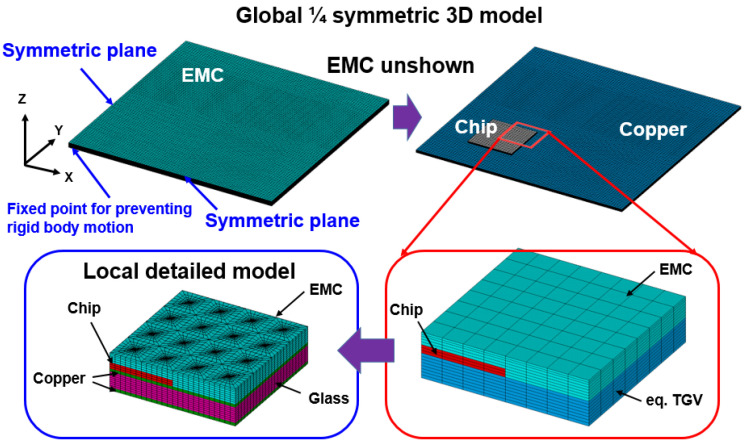
Flow chart diagram of the present submodeling simulation approach with the global interposer architecture model and local region model.

Submodeling techniques are a widely adopted modeling approach in simulation work, and their major contribution is to overcome modeling complexity and scaling mismatch between components in single FEA models. In the present study, the simulation work was performed by the commercial FEA software ANSYS 2019R1 version, and the utilized glass interposer architecture comprised a 100 mm × 100 mm interposer and microscaled Cu-filled TGV with 28-μm via diameter. For this reason, designing the decent mesh gridding of the constructed FEA model and extracting the converged numerical results by general simulation methodology were difficult. Accordingly, the submodeling technique was adopted to deal with the abovementioned issue. As illustrated in Figure 4, a one-quarter FEA model of an entire glass interposer vehicle was built in accordance with the symmetry of structure design and named as global model. Therefore, the inner side plane can be regarded as a symmetric plane (accordingly, the UX and UY displacement degree of freedom of left-sided plane and lower-sided plane were separately fixed), and all the displacement degrees of freedom on the bottom of the central axis were fixed to prevent the rigid body motion during simulation. In the global glass interposer model, the glass interposer within the TGV was modeled as a mixed equivalent material to economize the computing resource and improve the numerical convergence ability. Afterward, the region concerned was defined as the local model and constructed with detailed components; that is, the Si chip, glass interposer, Cu trace, and capped EMC in this study. The displacement field generated from the thermocompression process of the global glass interposer model was extracted and subsequently interpolated into the local model as the boundary conditions, and the thermocompression process-induced mechanical responses are introduced in the region concerned. Accordingly, the mechanical responses of the stress/strain distribution of the microscale TGV region can be effectively simulated with decent computing resources. 

**Figure 5:**
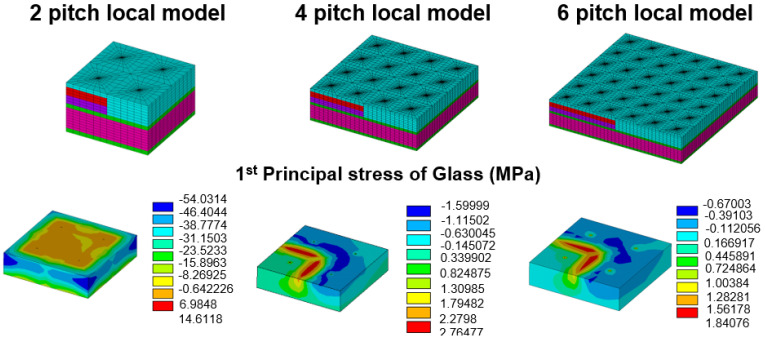
Numerical convergence analysis of the FEA submodeling local model with different via pitch amounts and the corresponding first principal stress distribution.

**Figure 6:**
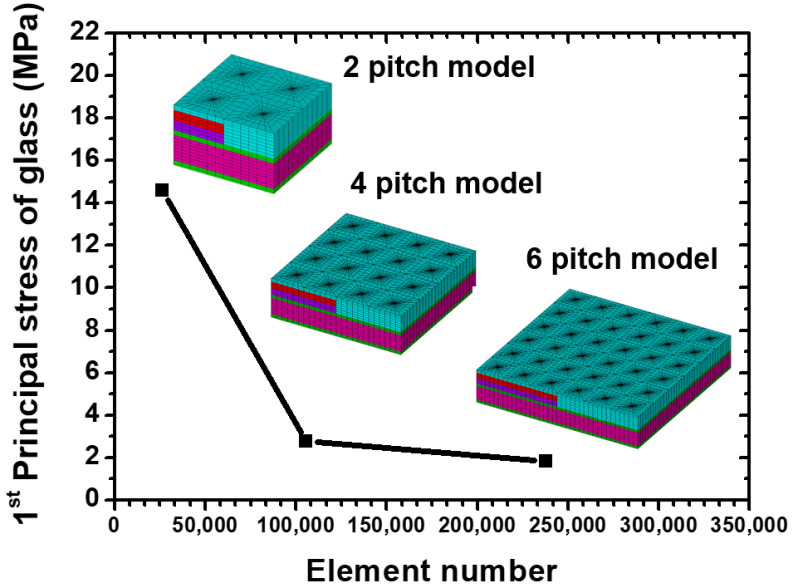
Maximum first principal stress of glass of local models with different via pitch amounts and element numbers.

Notably, the simulation results extracted by the submodeling technique should be checked carefully in accordance with Saint Venant’s principle. To prevent the influence of boundary condition on the stress/strain status of critical location, numerical convergence analysis was performed. The size of the local model was controlled by the different times of TGV pitch, and the critical first principal stress location and its magnitude were checked for stability and convergence. In Figure 5, the FEA model with different pitch and corresponding mesh gridding is presented, and the selected TGV pitch is defined as 2, 4, and 6. The boundary conditions of submodels were inserted into the displacement field of each plane extracted from the global TGV interposer model, and to observe the stress distribution away from the boundary conditions to prevent the stress concentration phenomenon near the boundary. In accordance with the first principal stress contour of two pitch local models, maximum stress was generated on the glass interposer. However, the critical stress location could not be observed clearly, and whether this phenomenon resulted from the adjacent boundary of the FEA model is unclear. To clarify this issue, the first principal stress contour of the four- and six-pitch models is also illustrated in Figure 5; the stress distribution and magnitude of the four- and six-pitch models was highly comparable, and the trend of convergence was expected. Finally, the maximum first principal stress of each model is summarized in Figure 6. About 14.61-, 2.76-, and 1.84-MPa first principal stresses were separately observed on the two-, four-, and six-pitch local model, and the corresponding element amount of the two-, four-, and six-pitch local models were counted as 26,400, 105,600, and 237,600, respectively. On the basis of the abovementioned analysis, the stability of the present submodeling approach was validated, and at least four TGV pitch-wide regions of the local model were needed to obtain the reliable stress distribution and magnitude. 

## 4. Results and Discussion

### 4.1. Thermocompression Process-Induced Stress Assessment of Glass with Various Chemical Shrinkage Extents

**Figure 7:**
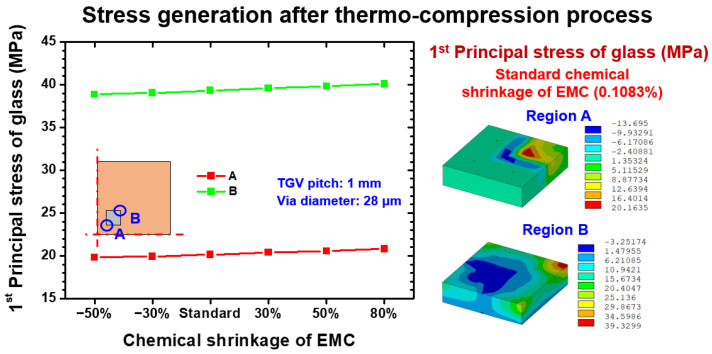
EMC shrinkage dependence first principal stress on the relevant location of the glass interposer after the thermocompression process.

As mentioned above, the mechanical responses introduced by thermocompression are the CTE mismatch thermal stress integrated with the chemical shrinkage of EMC itself. The CTE mismatch mechanism depends on the interactive effect of thermal mismatch deformation between material components in the vehicle. By contrast, the shrinkage behavior of EMC is relatively regulated by the composition of EMC, and the weight of shrinkage-induced stress in the entire thermocompression process is affected. To explore the influence of different shrinkage levels on the stress assessment of glass interposers, a parametric study that considered different levels of chemical shrinkage extent was performed. The chemical shrinkage of selected EMC under the fully cured condition was 0.1083%, the foregoing shrinkage was defined as the standard condition, and several variation levels (i.e., −50%, −30%, 30%, 50%, and 80%) were designed; the abovementioned percentage means the change based on the standard shrinkage amount. The maximum first principal stress of glass after fabrication at room temperature is summarized and illustrated in Figure 7. Two positions on the inner corner and outer corner of the Si chip, named as location A and B, were selected as the critical locations because the important CTE mismatch was generated between the Si chip and Cu trace (α = 3 ppm/K and α = 18 ppm/K) and between the Cu trace and glass interposer (α = 18 ppm/K and α = 0.52 ppm/K), causing potential risk of brittle fracture of the Si chip or glass interposer. Under different chemical shrinkage levels, the first principal stress of glass on locations A and B remained stable at 20 and 39 MPa, respectively. The first principal stress generated on location B was significantly larger than that on location A; this phenomenon can be explained by the distance from neutral point (DNP) effect. Accordingly, the harsher thermal deformation and corresponding first principal stress occurred on location B as compared with the status of location A. By contrast, the different levels of shrinkage did not affect the degree of first principal stress of the glass, probably because the glass interposer underneath the Cu trace and Si chip did not directly contact the capped EMC. Consequently, the additional loading introduced from the shrinkage of EMC did not influence the stress status of the glass interposer. 

**Figure 8:**
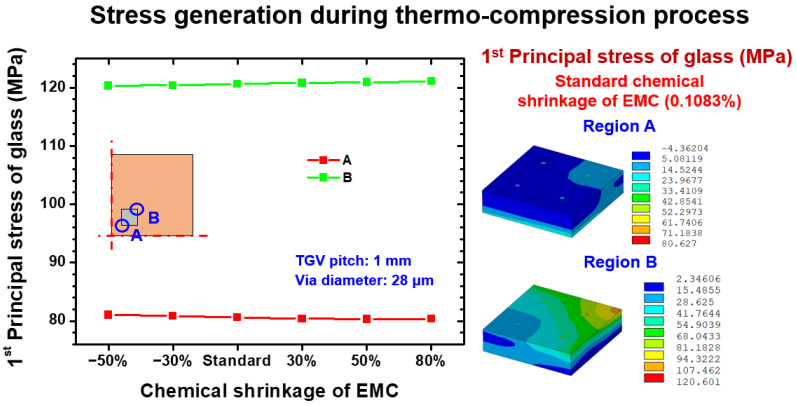
EMC shrinkage dependence first principal stress on the relevant location of the glass interposer during the thermocompression process.

To further investigate the mechanical reliability of the glass interposer during the fabrication process, the stress status of locations A and B under applied pressure of 5.5 kgf/cm^2^ (0.539 MPa) and 130 °C curing temperature loading are illustrated in Figure 8. Similarly to the trend presented in Figure 7, the introduced first principal stresses on location A and B were stable, and those stress magnitudes were 81 and 121 MPa, respectively. The different shrinkage deformation levels still had no considerable influence on induced stress. On the basis of the aforementioned analysis results, the integrated effect of applied pressure and raised temperature significantly enhanced the stress magnitude of the glass interposer. Given that the applied pressure mainly acted on capped EMC, its influence hardly affected the glass interposer through the Si chip and Cu trace. For this reason, the suddenly enlarged first principal stress of the glass was considered to be generated by the 130 °C curing temperature loading. Comparing the stress results of present study and Okoro’s data [12], it is evident that the thermocompression process-induced stress can reach up to 121 MPa under 130 °C curing temperature. In Okoro’s work, the TGV annealing procedure-induced stress on the glass interposer was 400 MPa under general 420 °C annealing temperature, and foregoing stress further enhanced the stress to 640 MPa with 650 °C annealing. It seems that the annealing procedure is also an important stress-generation mechanism of TGV interposer architecture and might cause the failure of the glass interposer. The comprehensive study on stress investigation of the glass interposer under annealing and thermocompression combined loading might should be explored in the future.

### 4.2. Thermocompression Process-Induced Stress Assessment of Si Chip with Various Chemical Shrinkage Extents 

**Figure 9:**
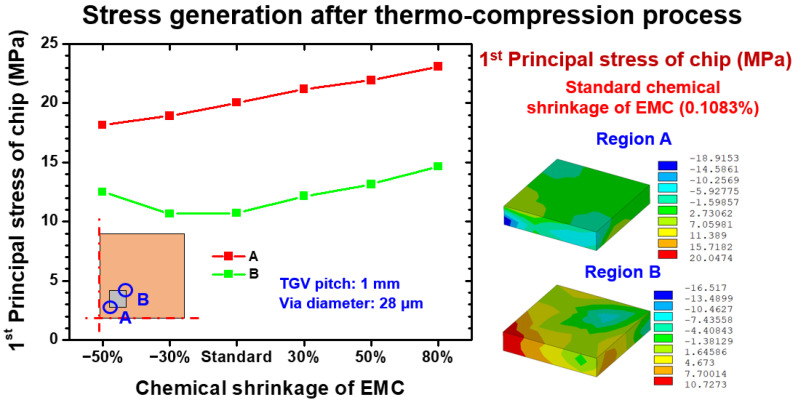
EMC shrinkage dependence first principal stress on the relevant location of the Si chip after the thermocompression process.

Another reliability issue is the potential risk of Si chip brittle fracture, especially the applied pressure in the thermocompression process and chemical shrinkage, which is easily imposed to the Si chip. The extracted first principal stresses on the inner and outer corners of the Si chip and corresponding stress distribution are illustrated in Figure 9. The simulated stress on location A was approximately linearly proportional to the increment of the EMC shrinkage level. However, the stress generated at location B was concentrated at the inside corner and was not purely dominated by DNP and the effect of shrinkage deformation. This characteristic is considered the consequence of the CTE mismatch stress discharged by the EMC shrinkage-induced stress at standard level. As the shrinkage level of EMC increased, the introduced first principal stress was subsequently enhanced, and the stress status of the observed location was dominated by the shrinkage deformation of EMC. Moreover, the influence of CTE mismatch deformation became the major stress-generation mechanism when the EMC shrinkage was suppressed below the standard shrinkage level of 0.1083%.

**Figure 10:**
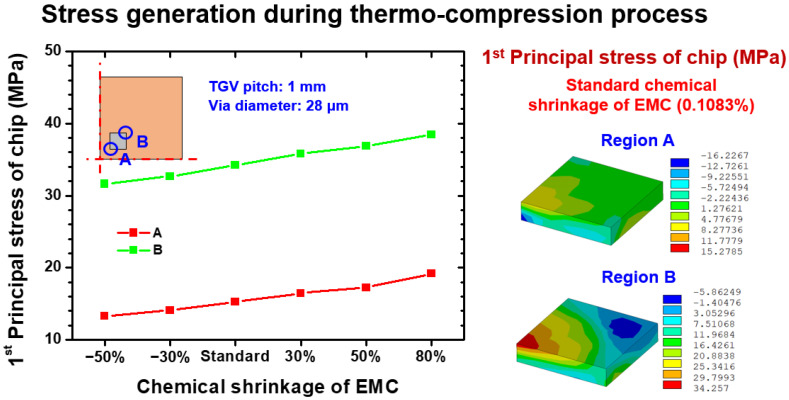
EMC shrinkage dependence first principal stress on the relevant location of the Si chip during the thermocompression process.

As shown in Figure 10, the first principal stress magnitude generated by the thermocompression process at 130 °C on locations A and B were linearly proportional to the chemical shrinkage level; this phenomenon is attributed to the shrinkage of EMC being directly applied on the Si chip and compressing the chip. Moreover, the EMC was found to have thermal stress-free status at 130 °C, indicating that the mechanical responses of EMC were dominated by chemical shrinkage. Notably, the critical stress location was observed on the inner corner of the Si chip (location A). This mechanism can be explained by the competition between CTE mismatch-induced stress and shrinkage-induced stress. For the stress status at the inner corner of the Si chip, the CTE mismatch stress was limited because the distance between location A and the neutral point of the entire glass interposer architecture was narrow, so that the corresponding DNP effect was not obvious. By contrast, the CTE mismatch stress on the outer corner of the Si chip (location B) had the same stress level as that of the shrinkage-induced stress. Accordingly, the stress status of location B remained as a lower stress condition, and the critical stress location was revealed as the inner corner of the Si chip. 

## 5. Conclusions

In this study, the mechanical responses of the thermocompression process generated on glass interposer architecture were analyzed using an FEA submodeling technique. Structural components of the Si chip and glass interposer and microscaled TGV were effectively integrated, and the stress responses of critical locations that had potential brittle failure risk were investigated. The numerical simulation accuracy and convergence stability of the present submodeling approach were validated by a parametric study with various local model regions. The analysis results revealed that at least four TGV pitch-wide regions of the local model were needed to obtain the converged and reliable simulation results. The present simulation methodology integrated with the process-oriented FEA simulation and submodeling technique, effectively conquered the complexity of the mesh gridding design of the TGV interposer architecture with significant size difference among components, and also obtained stable and reliable FEA results. After the validation of the present submodeling simulation approach, a parametric study, which considered the different levels of chemical shrinkage introduced from EMC material after the thermocompression process, was conducted. The stress distribution around the Si chip was observed because of the notable CTE mismatching between the Si chip and EMC, the Si chip and Cu trace, and the Cu trace and glass interposer. The maximum first principal stresses generated on the Si chip before and after thermocompression procedure were 39 MPa and 24 MPa, respectively. As a comparison, the first principal stresses on the TGV interposer separately induced before and after thermocompression were 120 MPa and 40 MPa. Based on the aforementioned simulated stresses and strength of Si and the glass itself, the major failure mode was considered as the brittle fracture of the glass interposer. The simulated results showed that the first principal stress generated on the glass interposer was dominated by the CTE mismatch mechanism rather than the shrinkage deformation of EMC. By contrast, the first principal stress observed on the Si chip was proportional to the amount of chemical shrinkage. Accordingly, the influence of two stress-generation mechanisms in the thermocompression process, the CTE mismatch and the curing process-induced shrinkage, were separately estimated via the demonstrated submodeling simulation approach.

## Data Availability

Data sharing is not applicable to this article.
